# A new densely-haired aroid species of *Homalomena* (Araceae) from North Sumatra, Indonesia

**DOI:** 10.3897/phytokeys.271.172410

**Published:** 2026-03-02

**Authors:** Muzzazinah, Inggar Damayanti, Muhammad Hisyam Fadhil, Erick Raynalta, Iin Pertiwi Amin Husaini, Reza Ramdan Rivai, Ade Agus Setiawan, Arifin Surya Dwipa Irsyam, Dian Rosleine, Mentari Putri Pratami, Muhammad Rifqi Hariri, Siti Roosita Ariati

**Affiliations:** 1 Biology Study Program, Faculty of Teacher Training and Education, Universitas Sebelas Maret, Surakarta 57126, Indonesia School of Life Sciences and Technology, Institut Teknologi Bandung Sumedang Indonesia; 2 Department of Forestry, Faculty of Agriculture, Universitas Lampung, Lampung 35111, Indonesia Universitas Indonesia Depok Indonesia; 3 Department of Biology, Faculty of Mathematics and Natural Science, IPB University, Bogor 16680, Indonesia Universitas Sebelas Maret Surakarta Indonesia; 4 Botani Tropika Indonesia Foundation (Botanika), Bogor 16113, Indonesia IPB University Bogor Indonesia; 5 Research Center for Applied Botany, National Research and Innovation Agency (BRIN), Cibinong 16911, Indonesia Universitas Lampung Lampung Indonesia; 6 Jungle Plants Farm, Bogor 16810, Indonesia Botani Tropika Indonesia Foundation (Botanika) Bogor Indonesia; 7 Herbarium Bandungense, School of Life Sciences and Technology, Institut Teknologi Bandung, Sumedang 45363, Indonesia Research Center for Applied Botany, National Research and Innovation Agency (BRIN) Cibinong Indonesia; 8 Research Center for Biosystematics and Evolution, National Research and Innovation Agency (BRIN), Cibinong 16911, Indonesia Jungle Plants Farm Bogor Indonesia; 9 Department of Biology, Faculty of Mathematics and Natural Science, Universitas Indonesia, Depok 16424, Indonesia Research Center for Biosystematics and Evolution, National Research and Innovation Agency (BRIN) Cibinong Indonesia

**Keywords:** *
Araceae
*, *
Chamaecladon
*, Indonesia, Malesia, Philodendreae

## Abstract

*Homalomena lingua-felis* is newly described from Tapanuli (North Sumatra Province, Indonesia). This species resembles *H. pexa* from which can be easily distinguished by its shorter petiole (2.0–4.5 cm vs. 7.0–12 cm), papillate petiole (vs. densely white-tomentose), elliptic or cordate to ovate leaf shape (vs. oblong- to ovate-cordiform), papillate abaxial leaf surface (vs. sparsely hairy), papillate veins (vs. tomentose), papillate peduncle and spathe (vs. tomentose), pendulous peduncle (vs. semi-erect to declinate), and conical staminate flower zone (vs. ellipsoid). This novelty represents a continuation of discoveries on hairy *Homalomena* in Sumatra.

## Introduction

The genus *Homalomena* Schott (Araceae Juss.) encompasses more than 160 species, which are found in tropical and subtropical areas of Asia, reaching into the South-west Pacific (POWO 2026). Members of *Homalomena* are distinguished by their aromatic vegetative tissue, an elongated spathe during the fruiting phase, a spathe free from the spadix, the absence of a spadix appendix, female flowers with a single staminode, and two to four free stamens in each male flower. Nevertheless, this genus displays significant morphological variation. Consequently, *Homalomena* has been delineated into four informal Supergroups (SGs): “*Chamaecladon*”, “*Cyrtocladon*”, “*Homalomena*”, and “*Punctulata*” (Wong et al. 2013).

The genus *Homalomena* comprises more than 30 distinct species native to Sumatra, Indonesia (Irsyam et al. 2025a, b; POWO 2026). The taxonomical study of Sumatran *Homalomena* is still in progress. The present subject has garnered significant attention from scholars in recent years, especially within the *Chamaecladon* Supergroup (SG). This SG consists of minute to small, frequently creeping herbs that rarely erect and are odorless or very rarely scented (Boyce and Wong 2008). The SG is recognized for its distinctive characteristics, including a small, oblong spathe that does not surpass two centimeters in length, and the presence of interpistillar staminodes that are shorter than the pistil. In addition, the staminate flowers in this SG have two stamens, with the connective not extending beyond the thecae (Wong et al. 2013, 2020a).

A number of recently delineated species within the *Chamaecladon*SG have been recorded in the last twelve years, as detailed by Boyce and Wong (2012, 2013, 2016a, b), along with Wong et al. (2020b). Some species exhibit intriguing pilose characteristics that render them particularly appropriate for decorative applications. This encompasses *H. hasei* P.C.Boyce & S.Y.Wong, *H. mobula* P.C.Boyce & S.Y.Wong, *H. pexa* S.Y.Wong, P.C.Boyce & A.Hay, and *H. squamis-draconis* P.C.Boyce & S.Y.Wong (Boyce and Wong 2016a, b; Wong et al. 2020b).

The island of Sumatra continues to present opportunities for the description of unrecognised species within the genus *Homalomena*. This manuscript presents a comprehensive account of a newly identified species of hairy *Homalomena* originating from Sumatra. The recently identified species, *H. lingua-felis* A.S.D.Irsyam, Raynalta, & M.R.Hariri, is classified within the *Chamaecladon*SG. This newly described species exhibits a striking resemblance to *H. pexa*, characterized by the presence of dense trichomes adorning the adaxial surface of the leaves. Our novelties showed that Sumatran *Homalomena* (*Chamaecladon*SG) exhibits an extensive range of morphological characteristics, indicating the presence of numerous unidentified species that may be documented in the future.

## Material and methods

### Plant collection and morphological observation

**Figure 1.**
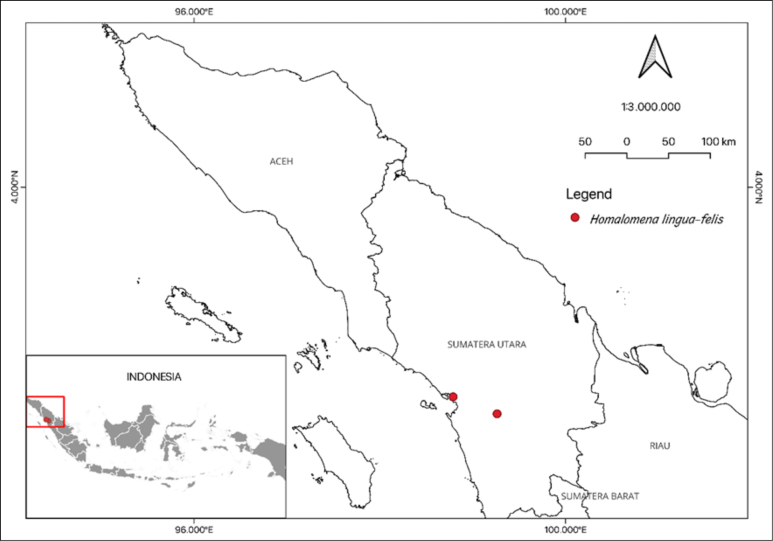
Study area of *Homalomena lingua-felis* in North Sumatra.

An expedition was conducted in Batang Toru, South Tapanuli, North Sumatra (Fig. 1), in January 2024. Plant materials were carefully collected, encompassing both preserved specimens and living collections. The morphological observations were carried out at the Laboratory for Plant Experiment - National Research and Innovation Agency (BRIN) and Herbarium Bandungense (FIPIA, code according to Thiers 2026 [continuously updated]), School of Life Science and Technology, ITB. In order to confirm the presence of identical specimens, further specimen examination was conducted at the Herbarium Bogoriense (BO)-BRIN and Singapore Botanic Gardens Herbarium (SING). In June 2024, we discovered several plant materials of the same species that had been cultivated in Jungle Plant Farm Nursery in Bogor Regency and Yagiza Nursery in Riau Regency. The terminology used in description follows Beentje (2016).

Conservation status of this new species was assessed using the IUCN Red List Categories and Criteria version 3.1 (IUCN 2024). The extent of occurrence (EOO) and area of occupancy (AOO) were calculated using GeoCAT (https://geocat.iucnredlist.org/) as described by Bachman et al. (2011).

### DNA extraction, amplification, and data analysis

Genomic DNA was isolated from fresh leaf tissues using the Tiangen Plant Genomic DNA Kit (TIANGEN Biotech, Beijing, China), in accordance with the manufacturer’s instructions. The DNA amplification was carried out in a total volume of 50 µL consisting of 10 ng DNA, 2 µM each for the internal transcribed spacer (ITS) 17SE-26SE primers (Sun et al. 1994), 17 μl ddH_2_O, and 25 µL MyTaq^TM^ HS Red Mix (Bioline, USA). The PCR profile used for amplification was a pre-denaturation at 94 °C for 5 mins; 35 cycles of denaturation at 94 °C for 15 s, annealing at 58 °C for 15 s, and extension at 72 °C for 15 s; final extension at 72 °C for 5 mins. The PCR product was submitted to 1^st^ Base (Malaysia) through PT. Genetika Science Indonesia for the sequencing process.

The ITS sequences of *H. lingua-felis* and *H. pexa* were individually assembled into contigs and jointly analyzed using NCBI BLAST (https://blast.ncbi.nlm.nih.gov/) to determine their sequence similarity. Sequence alignment of 43 ITS sequences from 42 species (Appendix 1) was performed using Clustal W in MEGA 12 (Kumar et al. 2024). Phylogenetic relationships were inferred using the Maximum Likelihood (ML) method implemented in IQ-TREE (https://iqtree.cibiv.univie.ac.at/; Trifinopoulos et al. 2016), with the best-fitting substitution model (TN+F+R2) selected by ModelFinder. We performed 1,000 bootstrap repeats to assess the reliability of the phylogenetic tree (Felsenstein 1985). The phylogenetic tree was visualized and annotated using Interactive Tree of Life website (https://itol.embl.de/, Letunic and Bork 2024). The ITS sequences of *H. lingua-felis* and *H. pexa* have been submitted to the NCBI under the accession numbers PQ591843 and PQ591844, respectively.

## Results

### Taxonomic treatment

#### Homalomena lingua-felis

Taxon classificationPlantaeAlismatalesAraceae

A.S.D.Irsyam, Raynalta & M.R.Hariri sp. nov.

5A43F401-AEAC-54D0-AAA2-DDBB3DBAE3D0

urn:lsid:ipni.org:names:77377370-1

Fig. 2

##### Type.

Indonesia • Sumatra, North Sumatra Province, South Tapanuli Regency, Batang Toru, 1°42'35.28"N, 98°48'49.68"E, c. 100 m, 2 Jan 2024, *E. Raynalta 02* (holotype FIPIA-DEP100!; isotype BO!).

**Figure 2.**
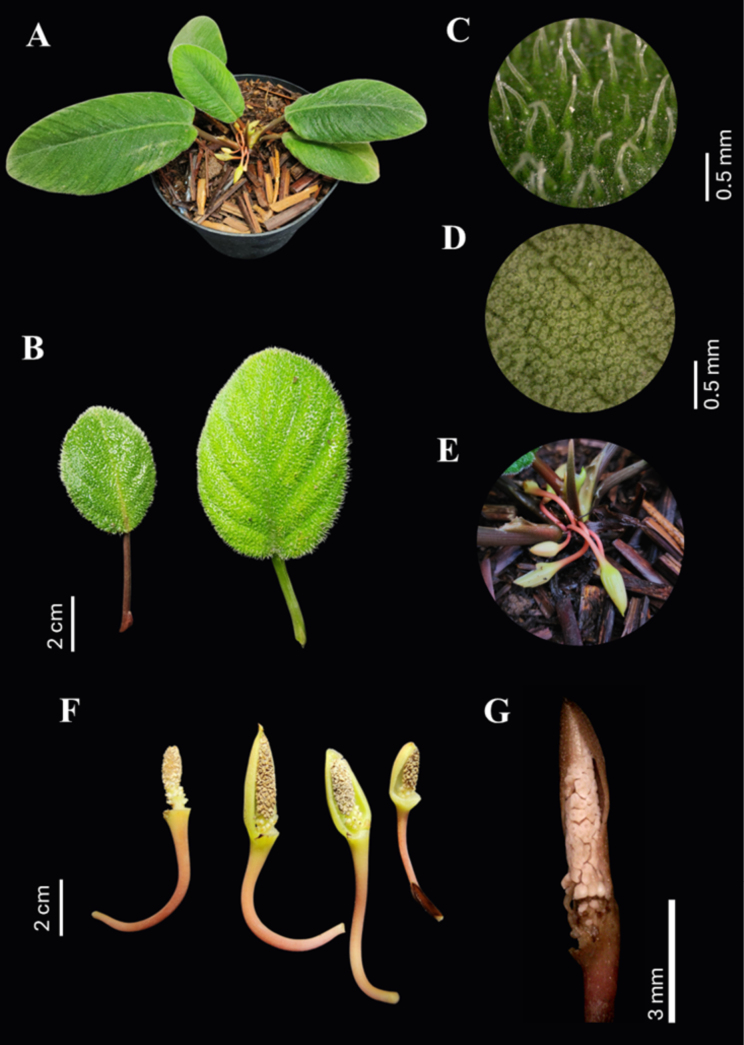
*Homalomena lingua-felis* A.S.D.Irsyam, Raynalta & M.R.Hariri, sp. nov. **A**. Habit; **B**. Adaxial surface of leaf showing dense hairs; **C**. Bulbous hair in adaxial leaf surface (59.4×); **D**. Papillose abaxial leaf surface (59.4×); **E**. Pendulous synflorescence; **F**. Bloom at late staminate anthesis with half and full spathe artificially removed; **G**. Bloom at late pistillate anthesis with half spathe artificially removed (20.7×). Photos by Erick Raynalta (**A, B, E**) and Muhammad Rifqi Hariri (**C, D, F, G**).

##### Diagnosis.

*Homalomena lingua-felis* is similar to *H. pexa* but differs by having shorter petiole (2.0–4.5 cm vs. 7.0–12 cm), papillate petiole (vs. densely white-tomentose), elliptic or cordate to ovate leaf shape (vs. oblong- to ovate-cordiform), papillate abaxial leaf surface (vs. sparsely hairy), papillate veins (vs. tomentose), papillate peduncle and spathe (vs. tomentose), pendulous peduncle (vs. semi-erect to declinate), and conical staminate flower zone (vs. ellipsoid).

##### Description.

Lithophytic herb to ca. 9.5 cm in height and 21 cm across. ***Stem*** condensed, ca. 2 cm long, ca. 0.5 cm in diam., red; internodes obscured by overlapping leaf bases. ***Leaves*** several together (3–7 per stem); petiole canaliculate, ribbed, terete, 1.2–4.5 cm long, 0.6–5.2 mm in diam., green reddish to dark red, papillose; petiolar sheath adnate to petiole, up to ½ the length of the petiole, 2.2–30.0 cm long, wings triangular, margin incurved, reddish-white; blade elliptic to cordate to ovate, subsucculent, 1.35–16.0 × 0.8–7.6 cm, base asymmetric cordate, margin ciliate, recurved, apex apiculate, adaxial surface green to dark green and densely tomentose hairs, hair bulbous, green at the base, white at the apex, abaxial surface greenish white, papillose; midrib prominent on abaxial surface, papillose, green or reddish; primary lateral veins 6–9 on each side diverging at 30°–50° from midrib, papillose, reddish abaxially. ***Inflorescences*** 3–7 together in a simple synflorescence, odorless, opening sequentially; peduncle pendulous, 6–8 mm long, inserted basally on spathe, pale red, papillose. ***Spathe*** 9.7–19.0 mm long, 3.7–5.5 mm wide across at the base, not constricted, exterior green or reddish brown to reddish-orange, interior green or creamy white, apex with a terminal mucro to 1–2 mm long, spathe opening by a rather wide slit, later closing and enclosing spadix. ***Spadix*** 7.1–15.4 mm long, stipitate; stipe ca. 0.5 mm long, creamy white; pistillate flower zone ca. ¼ the length of the spadix, 1.8–4 mm long; pistils in 2–3 whorls, loosely arranged, lageniform, 1.5–2.0 mm long, creamy white; ovary subglobose, ca. 1 mm long, white; stigma discoid, papillose; interpistillar staminode clavate, up to ca. 0.5 mm long, creamy white; staminate flower zone 5.3–11.4 mm long, narrowly conical, apex acute, creamy white; staminate flowers densely arranged, ca. 1 mm long, consisting of 2–4 stamens, trapezoid to hexagonal in plan view; thecae globose to ellipsoid, 0.5–0.7 mm long, white, opening by a wide terminal pore. ***Fruiting spathe, fruits* and *seeds*** not observed.

##### Etymology.

The specific epithet lingua-felis is derived from the Latin *lingua* (tongue) and *feles* (cat), in reference to the characteristic texture of the leaf surface, which bears a resemblance to the rough surface of a feline tongue.

##### Distribution and habitat.

**Figure 3.**
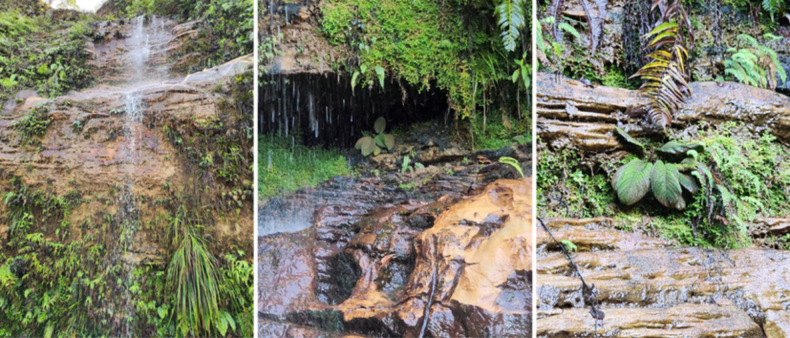
Habitat of *H. lingua-felis* in North Sumatra, Indonesia. Photos by Erick Raynalta.

*Homalomena lingua-felis* is naturally distributed in the regions of Central and South Tapanuli in North Sumatra. This study shows that *H. lingua-felis* has a narrow distribution, thus it can be stated as endemic to this area. The newly discovered species thrives on the vertical surfaces of stone walls found around waterfalls, at altitudes ranging from 15 to 50 m a.s.l. Our field study revealed that *H. lingua-felis* shared habitat with *H. anthurioides* S.Y.Wong, P.C.Boyce & A.Hay and *H. plicata* P.C.Boyce & S.Y.Wong in the same environment (Fig. 3).

##### Preliminary IUCN conservation assessment.

The species can be assessed as Vulnerable (VU) based on the criteria C1, C2(i), and D2, due to its restricted distribution (solely found in North Sumatra) with AOO and EOO of 8 km^2^, vulnerability of its habitat to fire, number of mature plants below 1,000 and high poaching activities due to its peculiar and attractive morphological characters, as displayed on various e-commerce platforms.

##### Additional specimens examined.

Indonesia • Sumatra, Riau, Rokan Hulu Regency, cultivated by Elis Nuraeni at Yagiza Nursery (original collection: Sumatra, North Sumatra, South Tapanuli, 2020, *E. Nuraini s.n*.), 0°31'27.408"N, 101°28'35.472"E, c. 12 m, 22 Jun 2024, *M. R. Hariri s.n*. (FIPIA-DEP101!). • Java, West Java, Bogor Regency, Pamijahan Subdistrict, cultivated by Ade Agus Setiawan at Jungle Plants Farm (original collection: Sumatra, North Sumatra, Central Tapanuli, Sibolga, 2019, *AA Setiawan s.n*.), 6°40'10.308"S, 106°40'40.764"E, c. 600 m, 8 Jun 2024, *M. R. Hariri & A. S. D. Irsyam s.n*. (FIPIA-DEP102!).

## Discussion

*Homalomena lingua-felis* is a remarkably attractive plant that has already been successfully cultivated by local (Indonesia) or foreign horticulturists. Interestingly, all of those cultivated materials can be traced back to the same locality, Tapanuli, North Sumatra. Several horticulturists frequently misidentify the new species as *H. pexa*, meanwhile the others mentioned the species as *Homalomena* “hairy” and due to its morphological similarity to the cat’s tongue, we propose the popular name for this species as *Homalomena* “lidah kucing”. Within its natural habitat, the species is susceptible to exploitation as a result of its ornamental value. In addition, the areas where this new species was discovered were impacted by a devastating forest fire in 2016. These factors could potentially increase the susceptibility of *H. lingua-felis* to extinction.

According to the previous studies, Asian *Homalomena* species have been divided into informal SGs: “*Homalomena*”, “*Chamaecladon*”, “*Cyrtocladon*”, and “*Punctulata*” (Boyce and Wong 2008; Ng et al. 2011; Wong et al. 2013). *H. lingua-felis* can be grouped as a member of the ChamaecladonSG based on its morphological characteristics, such as having a small and unconstricted spathe and a longer pistil in comparison to the interpistillar staminodes.

**Figure 4.**
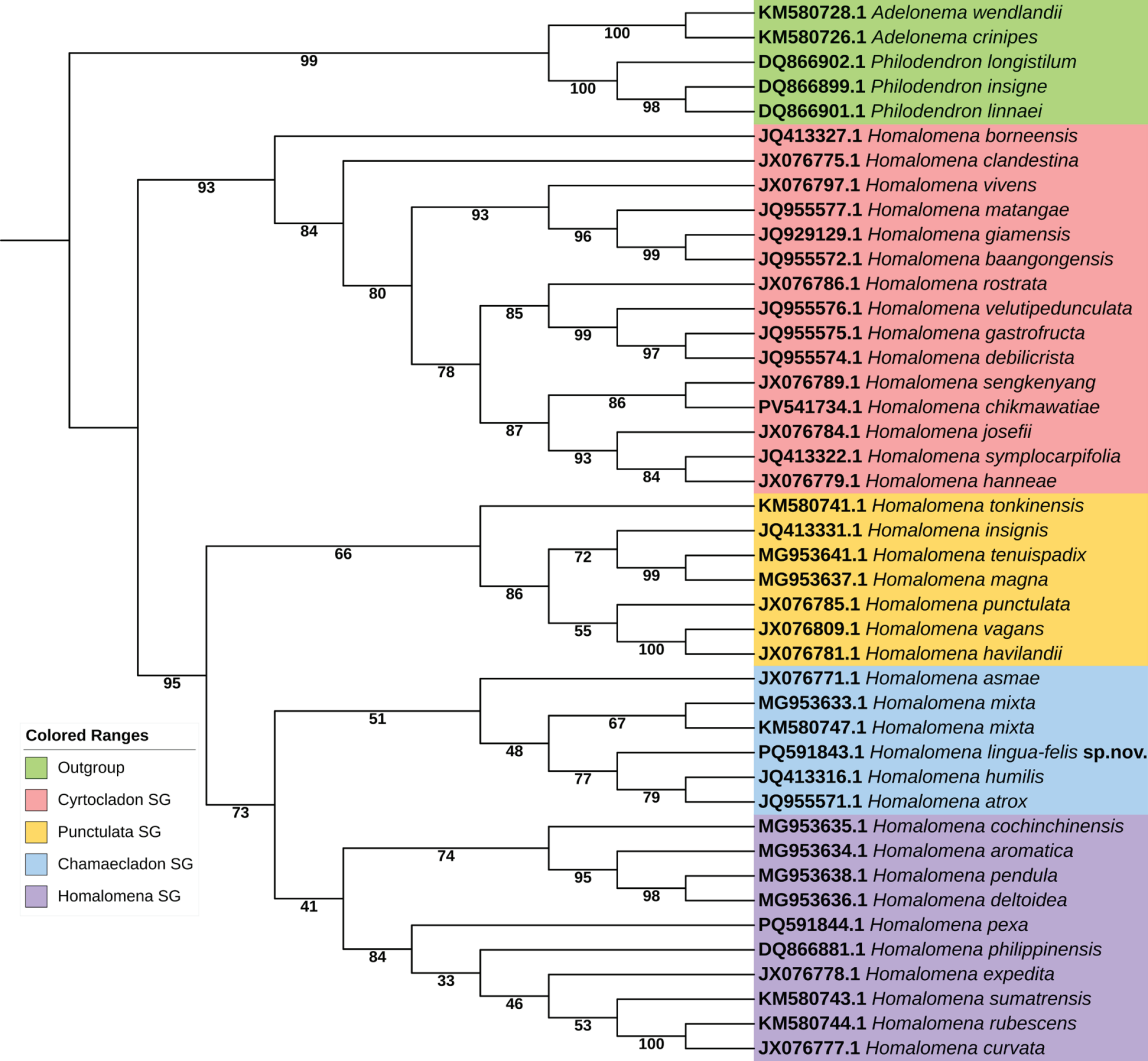
The placement of *Homalomena lingua-felis* within the phylogenetic tree based on ITS sequence using Maximum Likelihood method.

Phylogenetic analyses encompassed 42 taxa, including the neotropical species as outgroups from the genus *Philodendron* Schott and *Adelonema* Schott. The aligned data matrix is 978 base pairs in length, with 609 of these being constant. The informative character number for parsimony was 131 with log-likelihood value of BioNJ tree was -4049.802. The maximum likelihood analysis produced topologies for the examined *Homalomena* taxa (Fig. 4). The new species is classified in a distinct SG from *H. pexa*, which is morphologically similar, according to ITS regions. However, the new species is placed within the same ChamaecladonSG as the taxa analyzed in its corresponding supergroup.

Molecular analysis has elucidated the distinctiveness of *H. lingua-felis* and *H. pexa*, as depicted in Fig. 4. Both species, however, are classified within the Chamaecladon and HomalomenaSGs, respectively. Our findings align with those presented by Wong et al. (2020b). Although both species have been divided into distinct clusters, *H. lingua-felis* is taxonomically grouped with *H. atrox* P.C.Boyce, S.Y.Wong & Fasih and *H. humilis* (Jack) Hook.f., while *H. pexa* is associated with *H. philippinensis* Engl. and *H. expedita* A.Hay & Hersc. Phylogenetically, *H. lingua-felis* and *H. pexa* are distinct species, each exhibiting unique characteristics that differentiate them from one another supported by molecular approach.

The present study highlights the value of integrating molecular and morphological data in clarifying the phylogenetic relationships within *Homalomena*. Our findings demonstrate a general concordance between molecular identification and morphological taxonomy, indicating that DNA barcoding serves as a robust complementary tool to traditional morphological approaches. Although *H. pexa* has traditionally been assigned to the ChamaecladonSG based on morphological characters, our molecular analyses consistently place it within the HomalomenaSG. This incongruence suggests that morphology alone may not fully reflect evolutionary relationships in this lineage. One plausible explanation is morphological convergence, whereby similar vegetative or reproductive traits evolved independently in response to comparable ecological conditions rather than shared ancestry (James et al. 2023). Alternatively, the discordance may indicate cryptic speciation, in which genetically distinct lineages retain conserved morphological features, thereby obscuring true phylogenetic affinities (Stewart et al. 2014).

Similarly, a notable case is the occurrence of *H. rubescens* (Roxb.) Kunth—previously associated with a distinct supergroup—within the same SG as *H. pexa*, further complicating the delimitation of natural groupings based solely on morphology. Previous studies (Irsyam et al. 2025b) have also reported comparable inconsistencies, wherein *H. tonkinensis* Engl. (KM580741.1) and *H. cochinchinensis* Engl. (synonym of *H. occulta* (Lour.) Schott, MG953635.1), both unplaced in prior classifications, were molecularly grouped into the *Punctulata*SG and *Homalomena*SG, respectively. Collectively, these findings underscore the need for broader and more representative sequence data in future phylogenetic studies, as reliance on morphology alone may result in misleading classifications within *Homalomena*. Rather than weakening our conclusions, this incongruence highlights the complexity of evolutionary processes within the group and reinforces the importance of integrative taxonomic approaches that combine morphological evidence, molecular data, and expanded taxon sampling.

## Supplementary Material

XML Treatment for Homalomena lingua-felis

## Additional information

### Use of AI

No use of AI was reported.

### Author ORCIDs

Muzzazinah https://orcid.org/0000-0002-6862-2581

Inggar Damayanti https://orcid.org/0000-0002-7979-6931

Muhammad Hisyam Fadhil https://orcid.org/0000-0002-6329-8612

Erick Raynalta: https://orcid.org/0000-0002-3553-7735

Iin Pertiwi Amin Husaini https://orcid.org/0009-0005-5686-2667

Reza Ramdan Rivai https://orcid.org/0000-0002-8927-2429

Ade Agus Setiawan https://orcid.org/0009-0004-5743-4827

Arifin Surya Dwipa Irsyam https://orcid.org/0000-0001-7873-6665

Dian Rosleine https://orcid.org/0000-0002-8149-9629

Mentari Putri Pratami https://orcid.org/0000-0001-6282-2964

Muhammad Rifqi Hariri https://orcid.org/0000-0003-0943-3198

Siti Roosita Ariati https://orcid.org/0000-0001-8646-7055

### Data availability

All of the data that support the findings of this study are available in the main text.

## Appendix 1

**Table A1.** Species names and accession numbers included in the Maximum Likelihood phylogenetic analysis.

**Species**	**Collector**	**Collection number**	**Herbarium acronym**	**Barcode specimen number**	**Geographic origin**	**NCBI accession number**
*Adelonema wendlandii* (*Homalomena wendlandii*)	Wendland	*Wendland s. n.*.	SEL	1976-0060-009A	Costa Rica	KM580728.1
*Adelonema crinipes* (*Homalomena crinipes*)	T.B. Croat	*Croat 81956*	MO	-	-	KM580726.1
*Philodendron longistilum*		*Missouri Botanical Garden 11-17-78*	MO	-	-	DQ866902.1
*Philodendron insigne*	D. Barabe	*Barabe 75*	MT	-	-	DQ866899.1
*Philodendron linnaei*	D. Barabe	*Barabe 76*	MT			DQ866901.1
*Homalomena borneensis*	P. C. Boyce & al.	*AR2361*	-	-	Malaysia	JQ413327.1
*Homalomena clandestina*	Ng Kiaw Kiaw & Jipom Ak Tisai	*AR3007*			Malaysia	JX076775.1 H
*Homalomena vivens*	Ng Kiaw Kiaw & Jipom Ak Tisai	*AR3006*			Malaysia	JX076797.1
*Homalomena matangae*	P.C. Boyce & Jeland Ak Kisai	*AR230*	-	-	-	JQ955577.1 H
*Homalomena giamensis*	-	*-*	-	-	-	JQ929129.1 H
*Homalomena baangongensis*	P.C. Boyce & Wong Sin Yeng	*AR2574*	-	-	Malaysia	JQ955572.1
*Homalomena rostrata*	P.C. Boyce & Wong Sin Yeng	*AR2532*	-	-	Malaysia	JX076786.1
*Homalomena velutipedunculata*	P.C. Boyce & al.	*AR2103*	-	-	Malaysia	JQ955576.1
*Homalomena gastrofructa*	P.C. Boyce & Wong Sin Yeng	*AR2575*	-	-	Malaysia	JQ955575.1
*Homalomena debilicrista*	P.C. Boyce & Wong Sin Yeng	*AR3057*	-	-	Malaysia	JQ955574.1
*Homalomena sengkenyang*	P.C. Boyce & al.	*AR2388*	-	-	Malaysia	JX076789.1
*Homalomena chikmawatiae*	MR Hariri & ASD Irsyam	*MR Hariri & ASD Irsyam s.n*.	-	-	Indonesia: Riau	PV541734.1
*Homalomena josefii*	P.C. Boyce & al	*AR2380*	-	-	Malaysia	JX076784.1
*Homalomena symplocarpifolia*	P. C. Boyce & al.	*AR2411*	-	-	Malaysia	JQ413322.1
*Homalomena hanneae*	P.C. Boyce & al.	*AR2382*	-	-	Malaysia	JX076779.1
*Homalomena tonkinensis*	P.C. Boyce & S.Y. Wong	*P.C. Boyce & S.Y. Wong Ar4302*	-	-	Malaysia	KM580741.1
*Homalomena insignis*	P. C. Boyce & al.	*AR2066*	-	-	Malaysia	JQ413331.1
*Homalomena tenuispadix*	-	*564KG*	-	-	-	MG953641.1
*Homalomena magna*	-	*551KG*	-	-	-	MG953637.1
*Homalomena punctulata*	P.C. Boyce & al.	*AR2424*	-	-	Malaysia	JX076785.1
*Homalomena vagans*	Ng Kiaw Kiaw & Jipom Ak Tisai	*AR3011*	SAR	-	Malaysia	JX076809.1
*Homalomena havilandii*	P.C.Boyce & Jeland Ak Kisai	*AR2451*	-	-	Malaysia	JX076781.1
*Homalomena asmae*	Baharuddin	*Ar2597*	SAR	-	Malaysia	JX076771.1
*Homalomena mixta* (*Furtadoa mixta*)	C. Sakuragui	*C. Sakuragui 1402*	Living specimen	-	Malaysia	MG953633.1
*Homalomena mixta* (*Furtadoa mixta*)	Bogner s.n.	*Ar4781*	SAR	-	Malaysia	KM580747.1
*Homalomena lingua-felis*	*Erick Raynalta*	*E Raynalta 02*	FIPIA	FIPIA-DEP101	Indonesia	PQ591843.1
*Homalomena humilis*	P.C. Boyce & al.	*AR2371*	SAR	-	Malaysia	JQ413316.1
*Homalomena atrox*	P.C. Boyce & al.	*AR2389*	-	-	-	JQ955571.1
*Homalomena occulta* (*H. cochinchinensis*)	L. S. B. Calazans	*L. S. B. Calazans 36*	Living specimen	278RJ	-	MG953635.1
*Homalomena aromatica*	C. Sakuragui	*C. Sakuragui 1368*	-	554KG	-	MG953634.1
*Homalomena pendula*	C. Sakuragui	*C. Sakuragui 1372*	Living specimen	562KG	-	MG953638.1
*Homalomena deltoidea*	C. Sakuragui	*C. Sakuragui 1370*	-	548KG	-	MG953636.1
*Homalomena pexa*	*M. R. Hariri*	*M. R. Hariri s.n*.	Living specimen	-	-	PQ591844.1
*Homalomena philippinensis*	*-*	*Croat 52988*	MO	MBG 52988	-	DQ866881.1
*Homalomena expedita*	*P. C. Boyce*	*AR2357*	SAR	-	Malaysia	JX076778.1
*Homalomena sumatrensis* (*Furtadoa sumatrensis*)	*Nakamoto*	*Ar4045*	SAR	-	Indonesia	KM580743.1
*Homalomena rubescens*	*-*	*M527*	-	-	-	KM580744.1
*Homalomena curvata*	*P. C. Boyce & al*.	*Ar3053*	SAR	-	Malaysia	JX076777.1

Note: taxon names in parentheses correspond to the nomenclature used in the NCBI database.
